# Walking speeds are lower for short distance and turning locomotion: Experiments and modeling in low-cost prosthesis users

**DOI:** 10.1371/journal.pone.0295993

**Published:** 2024-01-02

**Authors:** Nidhi Seethapathi, Anil Kumar Jain, Manoj Srinivasan

**Affiliations:** 1 Brain and Cognitive Sciences, Massachusetts Institute of Technology, Cambridge, MA, United States of America; 2 Santokba Durlabhji Memorial Hospital, Jaipur, Rajasthan, India; 3 Mechanical and Aerospace Engineering, The Ohio State University, Columbus, OH, United States of America; National University of Ireland Galway, Galway, Ireland, IRELAND

## Abstract

Preferred walking speed is a widely-used performance measure for people with mobility issues, but is usually measured in straight line walking for fixed distances or durations, and without explicitly accounting for turning. However, daily walking involves walking for bouts of different distances and walking with turning, with prior studies showing that short bouts with at most 10 steps could be 40% of all bouts and turning steps could be 8-50% of all steps. Here, we studied walking in a straight line for short distances (4 m to 23 m) and walking in circles (1 m to 3 m turning radii) in people with transtibial amputation or transfemoral amputation using a passive ankle-foot prosthesis (Jaipur Foot). We found that the study participants’ preferred walking speeds are lower for shorter straight-line walking distances and lower for circles of smaller radii, which is analogous to earlier results in subjects without amputation. Using inverse optimization, we estimated the cost of changing speeds and turning such that the observed preferred walking speeds in our experiments minimizes the total cost of walking. The inferred costs of changing speeds and turning were larger for subjects with amputation compared to subjects without amputation in a previous study, specifically, being 4x to 8x larger for the turning cost and being highest for subjects with transfemoral amputation. Such high costs inferred by inverse optimization could potentially include non-energetic costs such as due to joint or interfacial stress or stability concerns, as inverse optimization cannot distinguish such terms from true metabolic cost. These experimental findings and models capturing the experimental trends could inform prosthesis design and rehabilitation therapy to better assist changing speeds and turning tasks. Further, measuring the preferred speed for a range of distances and radii could be a more comprehensive subject-specific measure of walking performance than commonly used straight line walking metrics.

## Introduction

**Fig 1:**
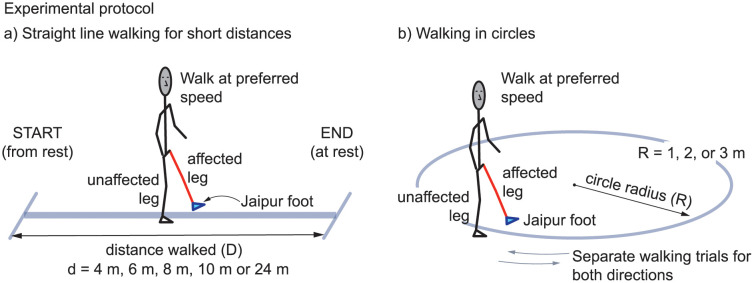
Overground walking experiment setup. We measured the preferred walking speed of walking for subjects with unilateral amputation wearing a Jaipur foot passive prosthetic leg in two conditions: a) walking a range of short distances, starting and stopping each bout at rest and b) walking in circles of different radii, both clockwise and anti-clockwise. These experiments have been previously performed in subjects without amputation [17, 52].

**Fig 2:**
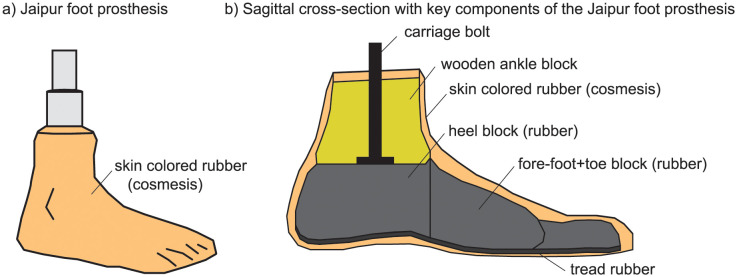
Jaipur foot prosthesis. a) Jaipur foot prosthesis has a skin colored cosmesis that simulates a barefoot appearance. b) Key components of the Jaipur foot prosthesis are shown. The interface between the various components have various rubber compounds for bonding and durability [77]. See [77, 78] for cross-sectional photographs, and [77] for more constructional details and measured mechanical properties.

Overground walking speed is commonly used to quantify a human subject’s mobility improvement after being fit with a new prosthetic leg or after undergoing physical therapy or rehabilitation from stroke, other injury, or movement disorder [1, 2]. Here, we study the preferred walking speeds for subjects with amputation while walking for short distances or while turning (Fig 1), showing that these speeds change systematically for shorter distances or higher curvatures of turning. We study these dependencies in people with unilateral transtibial amputation (TTA) and transfemoral amputation (TFA) in India wearing the Jaipur foot prosthesis (Fig 2). The Jaipur foot was developed by P. K. Sethi and co-workers in the 1970s [3], and is a low-cost prosthesis used widely in the developing world. It is used in over 22 countries and by hundreds of thousands of people [4, 5]. It is the second most widely used prosthesis after the SACH foot [4, 5]. This study adds to the small number of biomechanical studies on the Jaipur foot [6–9], which remains an under-studied prosthesis despite being so widely used.

Current state of the art walking speed tests include the ‘6 minute walk test’ [10, 11], which is framed implicitly as a cardiovascular endurance task with the subjects being asked to “walk as far as possible in the given duration,” and a ‘preferred walking speed’ test in which subjects walk short distances such as 3 m [12], 4 m [13], 5 m [14], 10 m [11] and 15 m [1, 15, 16] in a sub-maximal ‘comfortable’ or ‘natural’ manner. Table 1 summarizes variations of such speed measurements in the literature. In the current study, we focus on the latter version, where subjects with amputation walk naturally at their preferred walking speeds (also called ‘self-selected walking speeds’) for relatively short distances (Fig 1a). In healthy adults with no movement disorders, the preferred speed for walking in a straight line was recently shown to be distance-dependent [17], with the walking speed being systematically lower for shorter distances. In this study, we characterize this distance dependence of walking speeds in subjects with unilateral amputation wearing the Jaipur foot prosthesis. We propose that this distance dependence of walking speed could be a more complete measure of preferred walking speed, compared to measuring the speed at just one distance. This distance dependence of walking speed is further relevant because a considerable percentage of daily walking occurs in short bouts [18], especially in subjects with amputation [19, 20], with 40% of all bouts being 10 steps or less.

**Table 1:** Walking speed measurements over distance or time.

Distance (D) or Time (T) constrained	Distance	Time	References
D	3 m	-	[12, 21]
D	4 m	-	[13, 22]
D	5 m	-	[14, 23]
D	6 m	-	[21, 24]
D	8 m	-	[25, 26]
D	25 ft	-	[27, 28]
D	10 m	-	[13, 22, 29, 30]
D	12 m	-	[26]
D	15 m	-	[1, 15, 16]
D	50 ft	-	[31, 32]
D	20 m	-	[33, 34]
D	100 m	-	[27, 35]
D	400 m	-	[36, 37]
T	-	1 min	[38]
T	-	2 min	[30, 39–41]
T	-	6 min	[26, 30, 42, 43]
T	-	12 min	[26, 44]

Some previous studies that have measured walking speeds over different distances and time durations, but did not systematically study speed dependence on distance over multiple distances or radii as in this manuscript or in [17, 52]. In this list, the studies that prescribed a time duration did not constrain distance and the studies that prescribed a walking distance did not constrain time duration. See [45, 46] for studies that constrained both distance and time for other scientific purposes.

Effective mobility also requires ability to walk with turning [47]. Indeed, for subjects in one previous study, between 8% and 50% of all walking steps in daily life involved turning [48]. So, curved walking assessments could be useful in populations with locomotor challenges [49–51]. As a simple way of quantifying turning ability, we propose the measurement of preferred speeds while walking in circles of different radii (Fig 1b). We characterize such circle walking speeds in subjects with unilateral amputation. In adults without amputation, the tangential speed of walking was recently shown to depend on the curvature of the circle walked: slower walking for smaller circles or higher curvature [52]. The mechanics of subjects with amputation walking in a circle has been studied [20, 53, 54], but the radius dependence of preferred speeds has not previously been characterized.

Metabolic energy optimality has been used to make predictions for a number of aspects of overground and treadmill walking behaviors [55–57]. Preferred walking speeds in subjects with transfemoral amputation has been shown to be close to their energy optimal walking speeds by Esposito et al [58], expanding on a similar result by Ralston [59] in one subject. Here, we use energy optimization to interpret the distance-dependence of straight-line walking speed for short distances and the radius-dependence of walking speed in circles that we characterize in subjects with amputation. In subjects without amputation, the distance-dependence of straight-line walking speed was explained [17] by the larger energetic cost of speeding up and slowing down for shorter distances [17, 60, 61]. Similarly, in subjects without amputation, walking slower in tighter circles was explained [52] by the increased energetic cost of locomotion with turning during locomotion [52, 62]. By viewing preferred walking speeds in subjects with amputation through the lens of energy minimization, these short distance and circle walking tasks may provide insight into how the cost of changing speeds and the cost of turning may be affected by amputation. Walking with passive prosthetic legs usually results in a higher metabolic cost [63] than that in subjects without amputation, though recent work found no significant difference in some sub-populations with amputation [64]. It is not known whether subjects with amputation have higher energy costs of speeding up, slowing down, and turning when walking. Better understanding such components of energy cost in subjects with amputation may inform related work on prostheses aimed at reducing energy expenditure in walking [65–69] and understanding locomotor adaptation in populations with amputations or other movement disorders from an energetics perspective [65, 66, 69–72]. Here, instead of directly measuring the metabolic cost of changing speeds or turning in subjects with amputation, we estimate the cost of these components that will give rise to the observed average speeds. This process of determining the cost function, which when minimized gives the observed behavior, is sometimes called inverse optimization [73–75].

In summary, here, we examine the distance-dependence of preferred straight-line walking speeds and the radius-dependence of walking speeds while turning in subjects wearing the Jaipur foot prosthesis, performing human subject experiments to test the following three hypotheses: (1) that the average straight-line walking speed is lower for shorter distances, (2) that average walking speed is smaller for smaller radii while walking in circles, and (3) that subjects walk slower in circles when the prosthesis leg was on the inside. We found stronger evidence in favor of the first two hypotheses than the third. We then used inverse optimization methods to estimate the additional cost of changing speeds and turning that explains observed systematic trends in the data.

## Materials and methods

### Ethics statement

The experimental protocol was carried out in Jaipur, India. All subjects participated with verbal informed consent, as approved by the Ohio State University Institutional Review Board (IRB protocol 2012H0032) and the Santokba Durlabhji Memorial Hospital in Jaipur, India. Verbal informal consent was obtained by the experimenter by first explaining the procedures, risks, and benefits in the subjects’ native language (Hindi).

### Subject population

All subjects (*N* = 12 with 11 male, 1 female, 65.75 ± 12.6 kg with prosthesis and shoes, height 1.67±0.09 meters and age 39 ± 14.09 years, mean ± s.d.) had unilateral amputation: 7 subjects had transfemoral amputation and 5 subjects had transtibial amputation. In the rest of this manuscript, we refer to transfemoral amputation as above-knee amputation and transtibial amputation as below-knee amputation. The right leg was the affected leg for nine subjects and the left for three subjects. See Table 2 and S1 Data for individual subject data. All subjects had a unilateral Jaipur Foot prosthesis [3], either the above- or below-knee prosthesis. The subjects used the standard prosthesis that they wore every day and did not need to acclimatize to any new prostheses for the purposes of this study. All walking trials were also conducted at the Santokba Durlabhji Memorial Hospital in Jaipur. Inclusion criteria stipulated that the subjects be able to walk independently without using canes, crutches, hand rails, or assistive devices distinct from their prostheses. Each subject performed two kinds of walking trials: (1) walking for short distances (Fig 1a) and (2) walking in circles (Fig 1b), as described below. Between any two walking trials, subjects had breaks of durations between 15 seconds to up to a minute, with the break duration being self-selected by the subject. Subjects’ walking was video-recorded and time durations to complete a walking task were timed using the videos. The subjects did not carry any additional instrumentation. The subjects walked shod in these trials, using their daily foot-wear.

**Table 2:** Subject information.

Subject	Age	Mass	Height	*ℓ*_pros_	*ℓ*_intact_	BK/AK	Affected	Sex	*T*_onLegs_	*T*_amputation_
1	21	81	1.72	0.95	0.99	BK	L	M	3	5
2	40	55	1.54	0.99	0.96	AK	L	M	10	18
3	50	90	1.84	1.07	1.05	AK	L	M	10	30
4	35	66	1.77	0.98	0.99	AK	L	M	10	29
5	24	80	1.55	0.86	0.86	AK	R	F	5	14
6	38	60	1.71	1.00	0.99	AK	L	M	3	8
7	40	70	1.64	0.99	0.99	BK	L	M	3	17
8	53	64	1.63	1.02	1.02	AK	R	M	1	20
9	60	65	1.65	0.99	0.99	BK	L	M	2	23
10	22	47	1.67	1.05	1.05	AK	L	M	4	2
11	59	57	1.56	0.96	0.96	BK	L	M	1	9
12	26	54	1.74	1.01	1.00	BK	R	M	10	5

Age is in years. The height is with shoes on. The lengths *ℓ*_pros_ and *ℓ*_intact_ are, respectively, leg lengths on the prosthesis and intact sides. The height and these leg lengths are in meters. The column with BK or AK indicates whether the subject had below-knee or above-knee amputation, considered equivalent to trans-tibial or trans-femoral amputation here. The ‘affected’ column indicates whether the left (L) or the right (R) leg had the prosthesis. The duration *T*_onLegs_ indicates a self-reported time duration in hours that the subject spends each day on their legs, standing or walking. The duration *T*_amputation_ is the number of years since amputation.

### Jaipur foot prosthesis

The Jaipur foot prosthesis (Fig 2) was originally designed as a modification of the SACH foot [3, 76] by incorporating greater mobility in the ankle and subtalar joint for facilitating movements and postures common in India, such as bare foot or shod walking over unpaved uneven terrain and squatting or cross-legged sitting on the floor, allowing inversion and eversion [77]. It has three main pieces: a heel block and fore-foot-toe block made out of micro-cellular rubber bonded to a wooden ankle block (Fig 2b). Tread rubber is used on the undersurface for traction and durability, and the entire assembly is covered in a skin colored compound to provide cosmesis and water proofing. The Jaipur foot prostheses used in this study were manufactured and fit in the Santokba Durlabhji Memorial Hospital in Jaipur. The Jaipur foot prosthesis, not being patented, is manufactured by multiple local entities in different countries and sometimes even within the same country, essentially using an open source hardware model since the 1970s [77].

### Experiment: Walking for short distances

Each subject was instructed to walk in a straight-line for five different short distances *D*: 4 m, 6 m, 8 m, 10 m and 23 m (Fig 1a). There were four trials for each distance, resulting in 20 walking trials per subject. Trial order was randomized. Subjects were asked to “walk the way they usually walk” and they had to start and end each trial standing still, so they had to speed up from rest and slow down to rest. Average walking speeds over distances *D* were estimated by measuring the time duration *T* for each trial (starting and ending at rest) and computing the ratio (*D*/*T*) of the distance and time duration.

### Experiment: Walking in circles

Subjects were asked to walk in circles of three different radii: 1 m, 2 m and 3 m (Fig 1b), completing 5, 4 and 3 laps, respectively, for these radii. For each radius, subjects performed two trials: once with the prosthetic leg inside the perimeter and once with the prosthetic leg outside the perimeter of the circle—completing both clockwise and counter-clockwise trials. Trial order was randomized over the circle radii and walking directions. The average speed was obtained by measuring the total walking duration and averaging over all laps for each trial. Subjects walked with the circle between their two feet, maintaining a non-zero step width, rather than step on the circle with both feet.

### Hypothesis testing

We tested the following three *a priori* hypotheses:

1. **Average walking speed is lower for shorter distances**. For this distance-dependence test, we performed two types of tests: (1) We test whether each of the four shorter distance bouts (4, 6, 8, 10 m) are slower on average than the longer distance bout (23 m). This involves four tests, one for each distance, pooled across all subjects. (2) We fit a linear model to the shorter distance bouts (4–10 m) to see if the speed vs distance line has positive slope.
2. **Average walking speed is smaller for smaller radii while walking in circles**. For the radius-dependence experiment, we performed two types of tests: (1) We test whether walking on each of the three circles (1, 2, 3 m) are slower than walking in a straight line. This results in 3 tests, one for each radius, pooled across all subjects. For straight line walking, we used the longest distance we had measured, namely 23 m. (2) We fit a linear model to the circle walking speeds to see if the speed vs radius line has positive slope.
3. **Average circle walking speed is lower while walking with prosthetic side on inside of curve**. To test this hypothesis, we tested whether subjects walked slower in circles when the prosthesis leg was on the inside by pooling across all subjects with amputation and all radii.

To test these hypotheses, we used a within-subjects design (repeated measures), where the comparisons are performed between the subjects’ own speeds at different distances and different radii. We did not perform across-subjects or across-population comparisons. All hypothesis tests performed were paired (that is, within subject comparison and one-sided). The aforementioned linear models (speed vs distance and speed vs radius) use subject-specific offsets to account for the systematic speed differences between subjects. We performed the tests non-parameterically using bootstrap resampling with 10^5^ bootstrap samples [79–81]; this non-parametric approach allows for data complexity and non-normality due to the heterogeneous subject pool, accounting for correlated behavior for the different distances and radii by a given subject. The number of tests of statistical significance listed above is 10 and we simply used a Bonferroni correction to the *p* values to control for multiple comparisons. For comparison with the bootstrap procedure, we also provide one-sided t-test-based *p* values as well, also with Bonferroni correction. We label any additional statistical tests presented in the Results section as post hoc exploratory analysis.

### Mathematical model: Walking for short distances

For short distance walking, we compare the experimentally observed preferred walking speed results to the walking speed predicted by minimizing the total metabolic cost of the walking bout. We assume that people walking a distance *D* start from rest (as specified in experiment), then instantaneously speed up to some speed *v*, continue at that speed for the whole distance and then instantaneously come to rest again (following approach in [17]). Thus, we decompose the total metabolic cost *E*_total_ as a sum of two terms *E*_total_ = *E*_steady_ + *E*_acc−dec_, namely *E*_steady_ for walking at steady speed *v* and *E*_start−stop_ for accelerating from and decelerating to zero speed. This is a simple mathematical model meant to capture the primary costs of walking and changing speeds, while acknowledging that it ignores the more gradual changes in speed during gait initiation and termination for mathematical simplicity [82].

Walking steadily at constant speed *v* has previous been shown to have a metabolic rate ${\dot{E}}_{\text{steady}}$ that is well-approximated by a quadratic function of walking speed [56, 59, 83, 84], as follows:

$$\begin{array}{c} {\dot{E}}_{\text{steady}}=a_{0}+a_{1}v+a_{2}v^{2}. \end{array}$$ (1)

so that walking steadily for time duration *T* requires metabolic energy $E_{\text{steady}}={\dot{E}}_{\text{steady}}T$. Prior metabolic studies [59, 83, 84] characterized the coefficients *a*_0_, *a*_1_, *a*_2_ as follows: each study participant walked on a treadmill at a number of steady speeds *v* and the steady state metabolic rate $\dot{E}$ for each speed *v* was estimated through indirect calorimetry. Then, the coefficients *a*_0_, *a*_1_, *a*_2_ were estimated by fitting Eq 1 via ordinary least squares to the steady metabolic cost $\dot{E}$ versus speed *v* data. The values of these coefficients when metabolic rate ${\dot{E}}_{\text{steady}}$ is in W/kg and speed *v* is in m/s for subjects with above-knee amputation, with below-knee amputation, or without amputation from such prior studies are shown in Table 3. These relations for $\dot{E}$ result in a classical U-shaped relationship between energy cost per unit distance and speed of walking [56, 59, 84], as demonstrated in [58, 84] in subjects with amputation.

**Table 3:** Energy cost model coefficients.

Amputation	*a*_0_	*a*_1_	*a*_2_
Above-knee amputation	4.97	-5.98	5.62
Below-knee amputation	3.64	-2.19	2.89
None	2.22	0	1.115

Energy cost coefficients drawn from the literature for subjects with above-knee amputation [83], below-knee amputation [84], and no amputation [85]. The values and their units are consistent with metabolic rate $\dot{E}$ being in W/kg and speed in m/s.

Following [17], we modeled the acceleration and deceleration cost *E*_start−stop_ as proportional to the mechanical work necessary to achieve the corresponding changes in kinetic energy *mv*^2^/2 as follows:

$$E_{\text{start-stop}}=a_{\text{change}}(\frac{1}{\eta_{\text{pos}}}+\frac{1}{\eta_{\text{neg}}})(\frac{1}{2}mv^{2})$$

In this expression, the absolute kinetic energy change from rest to speed *v* or from speed *v* to rest is *mv*^2^/2. This kinetic energy change is scaled by the reciprocal of approximate muscle efficiencies for performing positive and negative mechanical work (*η*_pos_ = 0.25 and *η*_neg_ = 1.2 [86]) to obtain, respectively, an estimate of the metabolic cost of positive work of acceleration *mv*^2^/(2*η*_pos_) and the metabolic cost of negative work during deceleration *mv*^2^/(2*η*_neg_). We then multiply this estimate by an empirically derived scaling factor *a*_change_, previously suggested by Seethapathi and Srinivasan [17], who denoted it λ instead of *a*_change_ and estimated the metabolic cost of changing speeds by making subjects explicitly change walking speeds on an inertial frame and performing indirect calorimetry. Now that we have expressions for cost components *E*_steady_ and *E*_start−stop_, the total cost of the walking bout is given by:

$$\begin{array}{c} E_{\text{total}}=E_{\text{steady}}+E_{\text{start-stop}}=\left(a_{0}+a_{1}v+a_{2}v^{2}\right)\frac{D}{v}+a_{\text{change}}(\frac{1}{\eta_{\text{pos}}}+\frac{1}{\eta_{\text{neg}}})(\frac{1}{2}mv^{2}). \end{array}$$ (2)

The optimal speed *v*_opt_ that minimizes the short-distance cost of walking *E*_met_ is obtained by differentiating the total energy expression in Eq 2 and is given by the implicit equation:

$$\begin{array}{c} a_{\text{change}}v_{\text{opt}}^{3}({\eta_{\text{pos}}}^{-1}+{\eta_{\text{neg}}}^{-1})/\left(a_{0}-a_{2}v_{\text{opt}}^{2}\right)=D. \end{array}$$ (3)

This relation implies that shorter distance bouts should have lower optimal speeds [17].

### Mathematical model: Walking in circles

Brown et al [52] measured the steady state metabolic rate while walking in circles of different circle radii *R* and tangential speeds *v* for subjects without amputation via indirect calorimetry. They showed the metabolic rate of walking in a circle for subjects with amputation is well-described by a quadratic equation of the form: $\dot{E}=a_{0}+a_{1}v+a_{2}v^{2}+a_{\text{turn}}{\left(v/R\right)}^{2}$. The additional term *a*_turn_(*v*/*R*)^2^ compared to that for walking in a straight line (Eq 1) is the additional cost of turning. We posit that the same form for subjects with amputation, with *a*_0,1,2_ as used earlier in this section for subjects with above- or below-knee amputation [83, 84]. The cost per distance is given by:

$$\begin{array}{c} \dot{E}/v=a_{0}/v+a_{1}+a_{2}v+a_{\text{turn}}v/R^{2}, \end{array}$$ (4)

which is minimized by the following optimal speed [52]:

$$\begin{array}{c} v_{\text{opt}}=\sqrt{a_{0}/\left(a_{2}+a_{\text{turn}}/R^{2}\right)}. \end{array}$$ (5)

Thus, the prediction is that the optimal speed is smaller for smaller radius *R* [52].

For both short-distance walking and circle walking, we also determine the speeds that are within 1% of the minimum energy cost, because the energy landscapes are usually flat and a small change in speed near the minimum energy usually results in a much smaller energy change.

### Inverse optimization to estimate the cost of changing speeds and turning

We did not experimentally measure the metabolic energy costs of changing speeds (as in [17]) or turning (as in [52]) in subjects with amputation. Instead, we used inverse optimization to estimate the cost coefficients *a*_change_ and *a*_turn_ for changing speeds and turning respectively. Inverse optimization is a model-fitting procedure in which parameters governing the energy cost function are obtained in a manner that minimizing this energy cost predicts the experimentally observed behavior [73–75]. We performed two versions of the inverse optimization. In the first version, we held the steady state energy cost coefficients *a*_0_-*a*_2_ fixed and determined coefficients *a*_change_ and *a*_turn_ via inverse optimization. The inverse optimization is performed as follows: for fixed *a*_0−2_, *a*_change_ and *a*_turn_, we perform forward optimization analytically using Eqs 3–5 to compute the model-predicted optimal speeds and then compute the mean squared error (MSE) between predicted optimal speeds and observed preferred speeds: MSE = ∑(*v*_model_ − *v*_data_)^2^, averaged over all subjects and all trials. To determine the unknown coefficients, we used MATLAB’s fminunc to select the values of *a*_change_ and *a*_turn_ to minimize the MSE. This optimization procedure was validated by evaluating the MSE on a fine grid of *a*_change_ and *a*_turn_ values and selecting the lowest value of the MSE. This inverse optimization fit was performed separately for the subjects with above-knee and below-knee amputation.

In the second version of the inverse optimization, we solved for three coefficients, namely, *a*_2_, *a*_change_, and *a*_turn_. Though *a*_2_ is known from the literature (Table 3), here, we allowed *a*_2_ to be fit to the data to mitigate the risk of errors in the *a*_2_ values from literature (or being different from our participants’ *a*_2_ values) affecting the inferred *a*_change_ and *a*_turn_ values. We did not consider fitting *a*_1_ via inverse optimization because the Eqs 3–5 for the optimal speeds do not involve *a*_1_. We did not consider fitting *a*_0_ because the optimal speed expressions (Eqs 3–5) only involve ratios of the different coefficients, so allowing *a*_0_ to change will not reduce MSE further as the optimal ratios of coefficients can be obtained by just changing the other coefficients while keeping *a*_0_ fixed. Stated differently, the cost expressions in Eqs 2 and 4 can be multiplied by any constant value without affecting the optimal speeds, so this invariance to scaling allows us to fix one parameter arbitrarily. We chose to fix *a*_0_ and allow *a*_2_ to vary because *a*_2_ determines the velocity dependence of the straight line walking cost, and could accommodate other ‘costs’ perceived by the subject that may change with walking speed such as joint or interfacial stress, pain, stability, etc. Thus, a change in *a*_2_ from its nominal value in the literature could also be interpreted as a non-energy velocity dependent cost or benefit. 3–5 also indicate that while *a*_change_ only affects the short bout speeds and *a*_turn_ only affects circle walking speeds, *a*_2_ affects both short bout straight line walking and circle walking. So, the mean squared error (MSE) minimized by the inverse optimization is an average over both the short straight line bouts and the circle walking trials.

## Results

### Preferred walking speeds are lower for shorter distances

**Fig 3:**
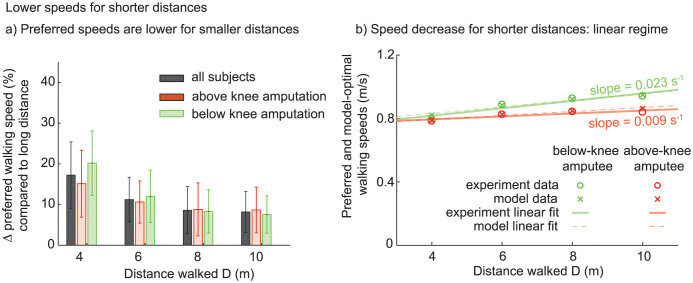
Decrease in preferred walking speed with distance walked for subjects with amputation. a) Subjects with amputation showed a decrease in average preferred walking speed for short distances. b) The rate of change in preferred walking speed with distance for the subjects with unilateral amputation is shown over a regime where both the subject-averaged data and the model fit are well-fit by linear trends (*R*^2^ value greater than 95%).

Subjects showed a decrease in the average preferred walking speed for shorter distances. Across all subjects, the preferred walking speed for each of the short distances (4 m, 6 m, 8 m and 10 m) was significantly lower than the preferred walking speed for the long-distance 23 m trial (each *p* < 10^−4^ using bootstrap and *p* ≤ 3 × 10^−3^ with left sided t-tests, all with Bonferroni corrections). The percentage decreases in the preferred walking speeds compared to the long distance 23 m trial, are shown in Fig 3a. On average, the percentage decrease in speed is larger for shorter distances: across all 12 subjects with amputation, the percentage decreases ranged from 8.2% ± 5% for the 10 m walk to 17.2% ± 8% for the 4 m walk (mean ± s.d.). In subjects with above-knee amputation, the percentage decreases ranged from 8.7% ± 5.6% for the 10 m walk to 15.1% ± 8% for the 4 m walk (mean ± s.d.). In subjects with below-knee amputation, the percentage decreases ranged from 7.5% ± 4.5% for the 10 m walk to 20% ± 8% for the 4 m walk. These results are summarized in Table 4.

**Table 4:** Speed reduction while walking for short bouts or in circles.

**a) Percent speed reduction over short straight-line bouts**			
Distance (D)	All subjects	Below-knee	Above-knee
10 m	8.2 ± 4.8% (*p* = 9 × 10^−4^)*	7.5 ± 4.1%*	8.7 ± 5.6%*
8 m	8.6 ± 5.5% (*p* = 4 × 10^−4^)^†^	8.3 ± 4.7%*	8.8 ± 6.0%^†^
6 m	11.2 ± 5.3% (*p* = 3 × 10^−3^)*	12.0 ± 5.8%*	10.6 ± 4.8%*
4 m	17.2 ± 7.8% (*p* = 2.6 × 10^−3^)*	20.2 ± 7.1%*	15.1 ± 7.6%*
**b) Percent speed reduction over circle walking**			
Radius (R)	All subjects	Below-knee	above-knee
3 m	11.4 ± 5.1% (*p* = 7 × 10^−5^)*	12.5 ± 5.4%*	10.6 ± 4.8%*
2 m	15.5 ± 5.5% (*p* = 7 × 10^−6^)*	17.2 ± 5.8%*	14.4 ± 5.0%*
1 m	30.2 ± 8.1% (*p* = 4 × 10^−7^)*	30.6 ± 10.1%*	30.0 ± 6.3%*

Percent reduction in speed is shown (mean ± s.d.) from the 23 m straight-line walking bout for: a) Short distance walking bouts for distances *D* = 4−10 m; b) Walking in circles. This table repeats information in Figs 3a and 5a.

* indicates that every subject in this condition had lower speed than the 23 m straight line bout.

^†^ indicates that in this condition exactly one subject had higher speed than the 23 m bout in exactly one trial.

Fitting a straight line to speed versus distance data for the short distance walking bouts, we find that the speed increases with distance with positive slope 0.015 s^−1^ (*p* = 3 × 10^−5^, fraction variance explained: adjusted *R*^2^ = 0.92). This confirms systematic increase in speed with distance. Separating out the subjects into above and subjects with below-knee amputation suggests a faster speed-increase with distance for subjects with below-knee amputation (Fig 3b), but we do not perform formal statistical comparison due to low subjects numbers.

### Increased cost of walking and changing speeds is consistent with speed-distance relationships

**Fig 4:**
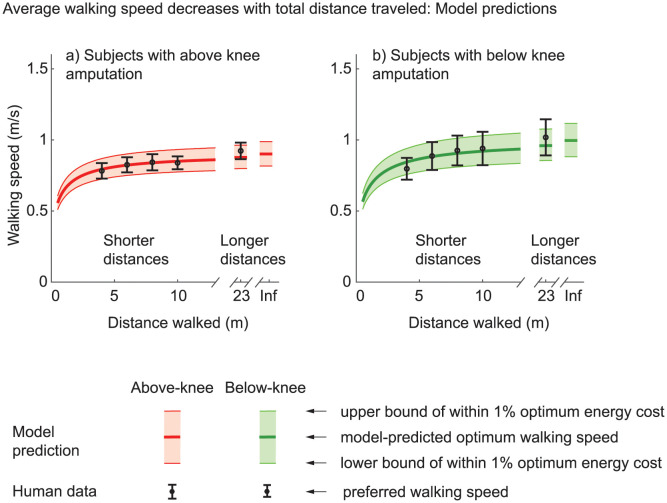
Minimization of total metabolic cost captures slower short-distance walking speeds. The total cost of the walking a short distance includes a term due to constant-speed cost and a changing-speed cost. We find that minimizing this total cost predicts the observed trends in changing preferred walking speed with distance for subjects with or without amputation. The error bars for human data represent standard errors, and the filled bands represent the set of all speeds within 1% of the energy optimal energy cost. The best fit cost coefficients obtained via inverse optimization shown in Table 5 are used here. The overall qualitative trends of smaller speeds for shorter distances remain as long as the steady walking cost coefficients *a*_0_ and *a*_2_, and the changing speed coefficient *a*_change_ are positive.

Our simple optimization-based model of short distance walking predicts the distance-dependence of optimal walking speed for both above and subjects with below-knee amputation (Figs 3 and 4), when we take into account the increased constant-speed metabolic cost of walking previously measured in experiments [83, 84] and an increased cost of changing speeds compared to subjects without amputation [17]. Using the first version of inverse optimization that kept *a*_2_ fixed, we estimated the coefficient *a*_change_ for the cost of changing speeds to be about 2.67 for subjects with below-knee amputation and 2.65 for subjects with above-knee amputation to best explain the observed walking speeds (Table 5), substantially higher than that for subjects without amputation [17]. Allowing *a*_2_ to change during inverse optimization in addition to *a*_change_ resulted in higher *a*_2_ values compared to that from literature (compare Tables 3 and 5). In this case, the inferred value of *a*_change_ was higher for subjects with above-knee amputation (1.84) compared to those with below-knee amputation (1.41), who had a slightly higher value than subjects without amputation (1.30). With the model coefficient values obtained from either version of inverse optimization, the mean preferred walking speeds plus one standard error is within 1% of the optimal energy costs from this model (Fig 4 and S5 Fig in S1 Appendix).

**Table 5:** Cost coefficients from inverse optimization.

Inverse optimization version 1			Inverse optimization version 2			
Amputation	*a*_change_	*a*_turn_	Amputation	*a*_2_	*a*_change_	*a*_turn_
Above-knee	2.67	7.71	Above-knee	6.12	1.84	6.53
Below-knee	2.65	6.64	Below-knee	3.66	1.41	4.26
None	1.34	1.11	None	1.19	1.30	1.04

Cost coefficients from inverse optimization to simultaneously fit the distance dependence of straight line walking speeds and the radius dependence of circle walking speeds. Versions 1 and 2 represent variants of the inverse optimization in which *a*_2_ is held fixed (version 1) or fit to the data (version 2). The quantities are consistent with $\dot{E}$ being in W/kg/s^2^ and speed in m/s. Results for subjects without amputation are from using [17, 52].

### Preferred walking speeds around circles are lower for smaller radii

**Fig 5:**
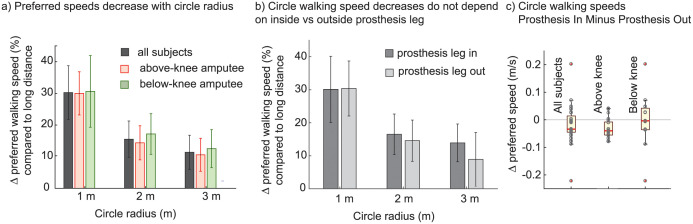
Preferred walking speeds for circle walking. a) The preferred walking speed for all the subjects with unilateral amputation showed a decrease with radius of the circle walked. b) Subjects with amputation, when pooled together, did not show a significant difference in preferred walking speed when walking with the prosthesis-leg inside versus outside the circle. c) Subjects with above-knee amputation show a greater walking speed on average when the prosthesis leg is outside the circle.

Subjects showed a decrease in preferred walking speeds in smaller circles. Pooled across all subjects, the preferred walking speed for each of the radii (1 m, 2 m, 3 m) was smaller than for the 23 m straight-line walking (each *p* < 10^−4^ using bootstrap and *p* < 10^−3^ using left sided t-tests, all with Bonferroni correction). See Table 4. Indeed, irrespective of type of amputation, all the subjects with unilateral amputation walked slower on circles on *every trial* compared to their straight-line walking trials (Fig 5a). This speed-dependence across different radii generalizes a previous comparison of walking speeds in a circle of a single radius with straight line walking [55]. Fitting a straight line between speed and radius with subject specific offsets, we get a slope of 0.09 s^−1^ (*p* < 10^−5^ and adjusted fraction variance explained, *R*^2^ = 0.92).

**Fig 6:**
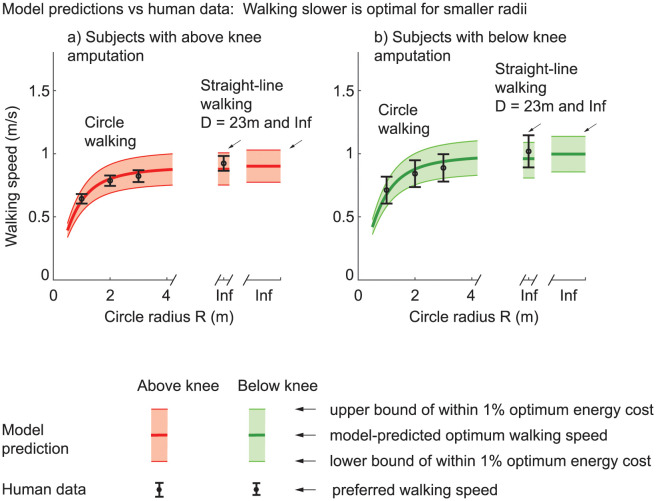
Optimal walking speeds for circle walking. Minimizing the energy cost of walking in a circle predicts slower walking for smaller circles. Error bars indicate one standard error about the mean, and these are generally within the set of all speeds within 1% of the optimal energy costs (the shaded bands shown). The cost coefficients used here are obtained via inverse optimization and shown in Table 5. This qualitative trend of lower speeds for smaller circles is predicted as long as the steady energy cost coefficients *a*_0_ and *a*_2_, and the turning cost coefficient *a*_turn_ are positive. At *R* → ∞ (Inf), the path is a straight line and the human speed data reported is for the longest bout performed (23 m).

Using inverse optimization to fit this speed dependence on circle radius by only changing the coefficient *a*_turn_ for the cost of turning, we find that the best-fit *a*_turn_ was about 6.6 W/kg/(s^−1^)^2^ more than that in subjects without amputation from [52] for subjects with below-knee amputation and about 7.7 W/kg/(s^−1^)^2^ for subjects with above-knee amputation (Table 5). Allowing *a*_2_ to change as well results in higher *a*_2_ than in the literature, while again resulting in the coefficient *a*_turn_ for turning to be higher for subjects with above-knee amputation (6.53) compared to below-knee amputation (4.26), which was in turn higher than subjects without amputation (1.04 [52]). The models from both inverse optimization versions contain the observed walking behavior inside the band of speeds that are within 1% of the optimal costs (Fig 6).

### Preferred walking speeds around circles is dependent on turning direction

We had subjects walk both clockwise and anti-clockwise along circles drawn on the ground. We did this so as to check for any effects due to having the prosthesis-leg as the pivot, as opposed to the intact leg as the pivot. Considering all subjects with amputation together, we did not find significant differences between the two conditions (*p* = 0.4, Fig 5b and 5c). However, as a post hoc exploratory test, just considering the subjects with above-knee amputation with trials for all radii pooled, we found that they walked slightly faster when the prosthetic leg was outside the circle (*v*_prosthesis−out_ − *v*_prosthesis−in_ = 0.03 ± 0.035 ms^−1^, *p* < 10^−3^; see Fig 5c).

### Preferred gait initiation swing is usually with the affected limb

As another post hoc exploratory analysis, we noted whether the subjects stepped forward with their affected or unaffected limb for their very first step. Stepping forward with the affected limb corresponds to the first swing phase being with the affected limb and the first stance phase being with the unaffected limb. We found that 9 out of 12 subjects had over 80% of their first steps be with their prosthetic foot; the other three subjects had 69%, 36%, and 0% of their steps start with swinging the affected limb. These leading limb preferences for gait initiation are similar to those found in [87].

## Discussion

Preferred walking speed is often used as a measure of progress in walking rehabilitation for various populations, for instance, persons with neuromuscular disorders and subjects with amputation [88], with faster speeds being considered better. Here, we found that subjects with unilateral amputation slow down on average when walking shorter distances, as did subjects without amputation [17]; see S1, S2 and S5 Figs in S1 Appendix for a visualization of data from subjects without amputation from [17] overlaid on Figs 3 and 4. We find that subjects with unilateral amputation also slow down when taking sharper turns (circles of smaller radii), analogous to subjects without amputation [52]; see S3-S5 Figs in S1 Appendix for a visualization of circle walking data from subjects without amputation from [52] overlaid on Figs 5 and 6. This speed dependence on the straight-line walking distance and the radius of turn may have implications for clinical practice, as discussed below.

In a clinical context, when quantifying a patient’s rehabilitation across time, if preferred speeds are measured only over a single distance, the distance must ideally be the same longitudinally across time (due to the distance dependence of speed)—as is likely in a given lab or hospital. In addition to such comparisons being only over the same fixed distance, we suggest measuring the speed over a few straight-line walking distances and a few circle-walking radii for each patient, not just one or two, thereby being able to map out a speed-dependence on distances and radii plot analogous to Figs 3–6. Detailed measurements may allow clinicians and scientists disambiguate ability for steady walking, gait initiation and termination, and turning. Using such speed dependence on distance and radius dependence may allow more robust comparisons of walking ability across time, across subject populations, and across labs, and would enable meta-analyses that pool information across studies conducted in different labs to examine efficacy of interventions. While multiple speed measurements over multiple distances and radii may require more time in the clinic for the patient, we estimate that the five distances and three radii used in this study could be completed in about 10 minutes even with over half a minute rest between trials. If the patient is limited in ability, it may be useful to restrict the trials to the shorter distances (up to 10 m, say) and shorter radii (1 m and 2 m, say), closer to the distances and radii used typically in normal walking [18, 48, 53].

We studied the preferred walking speeds of subjects with unilateral amputation wearing a particular passive prosthetic leg, namely, the Jaipur Foot prosthesis, used in a number of developing countries [3, 4, 6–9]. Using preferred walking speeds as a clinical performance measure may be more relevant where the resources available are limited, where access to other measures of performance, such as using a gait lab with motion capture and force plates, may be limited. The quantification of preferred walking speeds over multiple conditions (e.g., distances and radii as here) as a clinical measure would provide more information when walking-speed-based mobility measures are more exclusively used.

Most studies on energy optimality in locomotion involve constant-speed straight line walking on treadmills. Analogous to previous work on participants without amputation [17, 52], here, we have provided evidence that an optimality framework can predict aspects of non-steady or non-straight-line overground walking behavior in a population with amputation. The qualitative model predictions of slower speeds for smaller distances and slower speeds for smaller radii are true as long as the cost coefficient for changing speeds or turning is positive [17, 52].

Using inverse optimization to fit the short-distance walking model and circle-walking model to the measured walking speed behavior, we found that the coefficients for the cost of changing speeds and for the cost of turning (*a*_change_ and *a*_turn_, respectively in Eqs 2 and 4 respectively) are generally higher than for subjects without amputation. The changing speed coefficient *a*_change_ was higher for subjects with above-knee amputation in both versions of the inverse optimization, providing some confidence in the qualitative result. The estimated coefficient *a*_turn_ for the cost of turning was four to eight times larger for subjects with amputation compared to subjects without amputation; this trend was true in both versions of the inverse optimization. Thus, mechanistically, these values suggest larger costs for gait initiation, termination, and turning for subjects with amputation compared to the younger subjects without amputation in [17, 52]. Given the simplifying modeling assumptions, we can expect to be more confident of such qualitative results (e.g., in which population is the coefficient is larger), especially when the same qualitative result is obtained with different assumptions (e.g., the two versions of the inverse optimization), rather than the specific quantitative coefficient values obtained (e.g., a 4x to 8x increase in turning cost).

Because the coefficients *a*_2_, *a*_change_, and *a*_turn_ are obtained by inverse optimization to explain observed, they have the potential to include other non-energetic perceptual costs (or benefits) that affect the walking speeds, not manifested as energy costs. We found that the inverse optimization-inferred *a*_2_ was higher than that from the literature, which may either mean different subject characteristics from that in the literature or true additional velocity-dependent costs, possibly due to joint or interfacial stress or perceived stability. Such non-energetic costs may be true of the larger values for *a*_change_, and *a*_turn_ in subjects with amputation, although we may speculate that some of the increase is due to a reduced efficiency of propulsion in subjects with amputation. Stability considerations may be an important alternative to metabolic cost being the determinant of reduced speeds, especially for turning in a circle. One could examine this alternative hypothesis by having subjects use different speeds and estimating simple measures of stability [89, 90]. To test whether the increased cost coefficient estimates are entirely due to walking with a prostheses or if they may be confounded by age, we must repeat the experiments in age-matched subjects without amputation, while also controlling for any other confounders. To test whether these increased inverse optimization-derived cost predictions are indeed due to energy cost increases, one could directly measure the metabolic cost of changing speed and the cost of turning, as in [17, 52].

For short-distance walking, we measured only the average speed over the bout and not any other aspect of the speed changes. This average speed includes the acceleration and deceleration periods as characterized by Miff et al [82], who found that the acceleration and deceleration periods were about 1.5 to 1.7 seconds with a weak dependence on speed. So, the reduction in average speed is partly due to a greater portion of the bout being spent in acceleration-deceleration and partly due to reduced steady walking speed [17, 82, 91]. We did not measure the velocities during the acceleration-deceleration phases, their durations, or the steady walking speed. While we have assumed the steady walking speed to be the same as the average speed in the model, in reality, the steady or maximum walking speed will be higher than the average speed as it needs to compensate for the lower speeds during the acceleration-deceleration phases. The presence of more gradual acceleration-deceleration phases during typical walking [82] may suggest an additional energy cost penalty for speed change rates, not accounted for in this study [52]. Not including the gradual acceleration-deceleration phases may limit the quantitative accuracy of the cost of the changing speeds obtained via inverse optimization. To address this limitation, future work will involve characterizing the acceleration and deceleration phases as well as the full velocity trajectory, allowing us to test more detailed mathematical models. We note that this instantaneous acceleration assumption is not employed in our circle walking analyses, which require at least 30 m of walking over multiple laps so that a purely steady state walking analysis is appropriate [52].

We hypothesized a dependence of walking speed on turning direction, but found insignificant differences for across subjects with amputation and weak differences for just the subjects with above-knee amputation. We do not have a simple hypothesis for these small differences; understanding these differences may require a detailed biped model of how active push-off with the trailing leg helps with turning [52, 65, 92–94]. Optimization with biped models of subjects with amputation may also result in and predict the gradual acceleration and deceleration phases, as found in models of subjects without amputation [17, 91, 95]. Thus, such detailed biped models may also help mechanistically understanding the distance and radius dependence of walking speeds, as well as the differences between subjects with above-knee and below-knee amputation.

The sample size of subjects in this study was sufficient to show statistically significant differences for the specific hypotheses we tested, because of the within-subjects comparisons of speeds and repeated measures design of our study (analogous to prior studies [17, 52]). A larger and more diverse sample will allow us to observe the effect of other covariates such as body mass, age, sex, amputation level, height, time spent each day on their feet, and years since amputation. We hypothesize that the radius and distance dependence of walking speed may be quite general—that is, qualitatively similar across different populations but with potential quantitative differences. One of our goals here was to contribute to studying the Jaipur foot prosthesis in particular, given the disparity between the millions that use the device and paucity of quantitative biomechanical studies on using the device. Given the unique mechanical properties of the Jaipur foot, it would be valuable to repeat our study in other subject populations, specifically subjects with amputation wearing other prostheses with different mechanical properties.

One alternative to in-lab measurements during prescribed tasks is to track subjects’ speeds and movements all day using body-worn sensors such as pedometers, IMUs, GPS, etc. or video [48, 96–102] and repeating the analyses herein to such real-world data. Ultimately, these speeds during daily living may have greater relevance to quantifying mobility than lab-based measurements. Such ambulatory measurements provide an opportunity to independently corroborate the results in this study, by characterizing the speeds over bouts of different lengths and walks with turns that naturally occur during daily life. Such real world studies can also allow us to better choose the distances, turning radii, and tasks to be tested in in-lab conditions.

In conclusion, we have demonstrated that walking speed depends on the distance traveled for straight line walking and the turn radius while turning in subjects with amputation wearing the Jaipur foot prostheses. Given the relative simplicity of these tasks and given their potential ability to shed light on steady walking, turning, and gait initiation and termination, we propose that distance-dependence of walking speeds and radius-dependence of walking speeds be used as measures of mobility not just in subjects with amputation, but also other subject populations such as the elderly and those with or recovering from other movement disorders, when feasible.

## Supporting information

S1 Data Data from experiments. Compressed data file contains subject properties and the durations of walking bouts both for straight line and circle walking trials.
(ZIP)

S1 Appendix Additional figures. S1-S5 Figs in this appendix reproduces Figs 3 and 4, with data from subjects without amputation from [17, 52] re-drawn and overlaid for a qualitative visualization, and putting the results in this manuscript in the context of the older results.
(PDF)

## References

[1] Bohannon RW. Comfortable and maximum walking speed of adults aged 20 to 79 years: reference values and determinants. Age and ageing. 1997;26(1):15–19. doi: 10.1093/ageing/26.1.15 9143432

[2] Boonstra A, Fidler V, Eisma W. Walking speed of normal subjects and amputees: aspects of validity of gait analysis. Prosthetics and Orthotics International. 1993;17(2):78–82. doi: 10.3109/03093649309164360 8233772

[3] Sethi P, Udawat M, Kasliwal S, Chandra R. Vulcanized rubber foot for lower limb amputees. Prosthetics and orthotics international. 1978;2(3):125–136. doi: 10.3109/03093647809166697 733459

[4] Howitt P, Darzi A, Yang GZ, Ashrafian H, Atun R, Barlow J, et al. Technologies for global health. The Lancet. 2012;380(9840):507–535. doi: 10.1016/S0140-6736(12)61127-1 22857974

[5] Arya AP, Klenerman L. The jaipur foot. The Journal of bone and joint surgery British volume. 2008;90(11):1414–1421. doi: 10.1302/0301-620X.90B11.21131 18978257

[6] Arya A, Lees A, Nerula H, Klenerman L. A biomechanical comparison of the SACH, Seattle and Jaipur feet using ground reaction forces. Prosthetics and Orthotics International. 1995;19(1):37–45. doi: 10.3109/03093649509078230 7617457

[7] Lenka P, Kumar R. Gait comparisons of trans tibial amputees with six different prosthetic feet in developing countries. IPJMR. 2010; p. 8–14.

[8] Mishra P, Singh S, Ranjan V, Singh S, Pandey A, Mohanta M, et al. Performance evaluation of Jaipur knee joint through kinematics gait symmetry with unilateral transfemoral Indian amputees. Vibroengineering PROCEDIA. 2018;21:149–154. doi: 10.21595/vp.2018.20398

[9] Mishra P, Singh S, Ranjan V, Singh S, Vidyarthi A. Performance evaluation of Jaipur knee joint through kinematics and kinetics gait symmetry with unilateral transfemoral indian amputees. Journal of Medical Systems. 2019;43(3):55. doi: 10.1007/s10916-019-1181-0 30694396

[10] Harada ND, Chiu V, Stewart AL. Mobility-related function in older adults: assessment with a 6-minute walk test. Archives of physical medicine and rehabilitation. 1999;80(7):837–841. doi: 10.1016/S0003-9993(99)90236-8 10414771

[11] Amatachaya S, Naewla S, Srisim K, Arrayawichanon P, Siritaratiwat W. Concurrent validity of the 10-meter walk test as compared with the 6-minute walk test in patients with spinal cord injury at various levels of ability. Spinal Cord. 2014;52(4):333. doi: 10.1038/sc.2013.171 24445972

[12] Graham JE, Ostir GV, Fisher SR, Ottenbacher KJ. Assessing walking speed in clinical research: a systematic review. Journal of evaluation in clinical practice. 2008;14(4):552–562. doi: 10.1111/j.1365-2753.2007.00917.x 18462283 PMC2628962

[13] Peters DM, Fritz SL, Krotish DE. Assessing the reliability and validity of a shorter walk test compared with the 10-Meter Walk Test for measurements of gait speed in healthy, older adults. Journal of geriatric physical therapy. 2013;36(1):24–30. doi: 10.1519/JPT.0b013e318248e20d 22415358

[14] Wilson CM, Kostsuca SR, Boura JA. Utilization of a 5-meter walk test in evaluating self-selected gait speed during preoperative screening of patients scheduled for cardiac surgery. Cardiopulmonary physical therapy journal. 2013;24(3):36. doi: 10.1097/01823246-201324030-00006 23997690 PMC3751713

[15] Dobkin BH. Short-distance walking speed and timed walking distance: redundant measures for clinical trials? Neurology. 2006;66(4):584–586. 16505318 10.1212/01.wnl.0000198502.88147.dd

[16] Dickstein R. Rehabilitation of gait speed after stroke: a critical review of intervention approaches. Neurorehabilitation and neural repair. 2008;22(6):649–660. doi: 10.1177/1545968308315997 18971380

[17] Seethapathi N, Srinivasan M. The metabolic cost of changing walking speeds is significant, implies lower optimal speeds for shorter distances, and increases daily energy estimates. Biology letters. 2015;11(9):20150486. doi: 10.1098/rsbl.2015.0486 26382072 PMC4614425

[18] Orendurff MS, Schoen JA, Bernatz GC, Segal AD, Klute GK. How humans walk: bout duration, steps per bout, and rest duration. Journal of Rehabilitation Research & Development. 2008;45(7). 19165696 10.1682/jrrd.2007.11.0197

[19] Klute GK, Berge JS, Orendurff MS, Williams RM, Czerniecki JM. Prosthetic intervention effects on activity of lower-extremity amputees. Archives of physical medicine and rehabilitation. 2006;87(5):717–722. doi: 10.1016/j.apmr.2006.02.007 16635636

[20] Shell CE, Segal AD, Klute GK, Neptune RR. The effects of prosthetic foot stiffness on transtibial amputee walking mechanics and balance control during turning. Clinical Biomechanics. 2017;49:56–63. doi: 10.1016/j.clinbiomech.2017.08.003 28869812

[21] Lyons JG, Heeren T, Stuver SO, Fredman L. Assessing the agreement between 3-meter and 6-meter walk tests in 136 community-dwelling older adults. Journal of aging and health. 2015;27(4):594–605. doi: 10.1177/0898264314556987 25376604 PMC4522919

[22] Unver B, Baris RH, Yuksel E, Cekmece S, Kalkan S, Karatosun V. Reliability of 4-meter and 10-meter walk tests after lower extremity surgery. Disability and rehabilitation. 2017;39(25):2572–2576. doi: 10.1080/09638288.2016.1236153 27728985

[23] Fulk GD, Echternach JL, Nof L, O’Sullivan S. Clinometric properties of the six-minute walk test in individuals undergoing rehabilitation poststroke. Physiotherapy theory and practice. 2008;24(3):195–204. doi: 10.1080/09593980701588284 18569856

[24] Roush J, Heick JD, Hawk T, Eurek D, Wallis A, Kiflu D. Agreement in Walking Speed Measured Using Four Different Outcome Measures: 6-Meter Walk Test, 10-Meter Walk Test, 2-Minute Walk Test, and 6-Minute Walk Test. Internet Journal of Allied Health Sciences and Practice. 2021;19(2):7.

[25] Krumpoch S, Lindemann U, Becker C, Sieber CC, Freiberger E. Short distance analysis of the 400-meter walk test of mobility in community-dwelling older adults. Gait & Posture. 2021;88:60–65. doi: 10.1016/j.gaitpost.2021.05.002 34000486

[26] Eng JJ, Chu KS, Dawson AS, Kim CM, Hepburn KE. Functional walk tests in individuals with stroke: relation to perceived exertion and myocardial exertion. Stroke. 2002;33(3):756–761. doi: 10.1161/hs0302.104195 11872900

[27] Phan-Ba R, Pace A, Calay P, Grodent P, Douchamps F, Hyde R, et al. Comparison of the timed 25-foot and the 100-meter walk as performance measures in multiple sclerosis. Neurorehabilitation and neural repair. 2011;25(7):672–679. doi: 10.1177/1545968310397204 21436388

[28] Larson RD, Larson DJ, Baumgartner TB, White LJ. Repeatability of the timed 25-foot walk test for individuals with multiple sclerosis. Clinical rehabilitation. 2013;27(8):719–723. doi: 10.1177/0269215512470269 23426567

[29] van Hedel HJ, Dietz V, Curt A. Assessment of walking speed and distance in subjects with an incomplete spinal cord injury. Neurorehabilitation and neural repair. 2007;21(4):295–301. doi: 10.1177/1545968306297861 17353459

[30] Chan WL, Pin TW. Reliability, validity and minimal detectable change of 2-minute walk test, 6-minute walk test and 10-meter walk test in frail older adults with dementia. Experimental gerontology. 2019;115:9–18. doi: 10.1016/j.exger.2018.11.001 30423359

[31] Özden F, Özkeskin M, Bakırhan S, Şahin S. The test–retest reliability and concurrent validity of the 3-m backward walk test and 50-ft walk test in community-dwelling older adults. Irish Journal of Medical Science (1971-). 2022;191(2):921–928. doi: 10.1007/s11845-021-02596-1 33715071

[32] Unver B, Kalkan S, Yuksel E, Kahraman T, Karatosun V. Reliability of the 50-foot walk test and 30-sec chair stand test in total knee arthroplasty. Acta ortopedica brasileira. 2015;23:184–187. doi: 10.1590/1413-78522015230401018 26327798 PMC4544525

[33] Motyl JM, Driban JB, McAdams E, Price LL, McAlindon TE. Test-retest reliability and sensitivity of the 20-meter walk test among patients with knee osteoarthritis. BMC musculoskeletal disorders. 2013;14(1):1–8. doi: 10.1186/1471-2474-14-166 23663561 PMC3661341

[34] Øiestad BE, White DK, Booton R, Niu J, Zhang Y, Torner J, et al. Longitudinal course of physical function in people with symptomatic knee osteoarthritis: data from the multicenter osteoarthritis study and the osteoarthritis initiative. Arthritis care & research. 2016;68(3):325–331. doi: 10.1002/acr.22674 26236919 PMC4879777

[35] Belachew S, CALAY P, DELVAUX V, HYDE R, Hottermans C, Moonen G. The timed 100-meter walk test: an easy-to-use, sensitive tool to detect and evaluate restricted walking capacities in multiple sclerosis. In: 19th Meeting of the European Neurological Society; 2009. p. 17–20.

[36] Vestergaard S, Patel KV, Bandinelli S, Ferrucci L, Guralnik JM. Characteristics of 400-meter walk test performance and subsequent mortality in older adults. Rejuvenation research. 2009;12(3):177–184. doi: 10.1089/rej.2009.0853 19594326 PMC2939839

[37] Gabriel KKP, Rankin RL, Lee C, Charlton ME, Swan PD, Ainsworth BE. Test-retest reliability and validity of the 400-meter walk test in healthy, middle-aged women. Journal of Physical Activity and Health. 2010;7(5):649–657. doi: 10.1123/jpah.7.5.649 20864761

[38] McDowell BC, Kerr C, Parkes J, Cosgrove A. Validity of a 1 minute walk test for children with cerebral palsy. Developmental medicine and child neurology. 2005;47(11):744–748. doi: 10.1111/j.1469-8749.2005.tb01071.x 16225737

[39] Bohannon RW. Normative reference values for the two-minute walk test derived by meta-analysis. Journal of physical therapy science. 2017;29(12):2224–2227. doi: 10.1589/jpts.29.2224 29643611 PMC5890237

[40] Brooks D, Parsons J, Hunter JP, Devlin M, Walker J. The 2-minute walk test as a measure of functional improvement in persons with lower limb amputation. Archives of physical medicine and rehabilitation. 2001;82(10):1478–1483. doi: 10.1053/apmr.2001.25153 11588757

[41] Reid L, Thomson P, Besemann M, Dudek N. Going places: does the two-minute walk test predict the six-minute walk test in lower extremity amputees? Journal of rehabilitation medicine. 2015;47(3):256–261. doi: 10.2340/16501977-1916 25588644

[42] Enright PL, McBurnie MA, Bittner V, Tracy RP, McNamara R, Arnold A, et al. The 6-min walk test: a quick measure of functional status in elderly adults. Chest. 2003;123(2):387–398. doi: 10.1378/chest.123.2.387 12576356

[43] Chetta A, Zanini A, Pisi G, Aiello M, Tzani P, Neri M, et al. Reference values for the 6-min walk test in healthy subjects 20–50 years old. Respiratory medicine. 2006;100(9):1573–1578. doi: 10.1016/j.rmed.2006.01.001 16466676

[44] Campo LA, Chilingaryan G, Berg K, Paradis B, Mazer B. Validity and reliability of the modified shuttle walk test in patients with chronic obstructive pulmonary disease. Archives of physical medicine and rehabilitation. 2006;87(7):918–922. doi: 10.1016/j.apmr.2006.03.005 16813778

[45] Long LL III, Srinivasan M. Walking, running, and resting under time, distance, and average speed constraints: optimality of walk–run–rest mixtures. Journal of The Royal Society Interface. 2013;10(81):20120980. doi: 10.1098/rsif.2012.0980 23365192 PMC3627106

[46] Tiew EH, Seethapathi N, Srinivasan M. Pre-crastination: time uncertainty increases walking effort. bioRxiv. 2020;.

[47] Gailey RS, Roach KE, Applegate EB, Cho B, Cunniffe B, Licht S, et al. The amputee mobility predictor: an instrument to assess determinants of the lower-limb amputee’s ability to ambulate. Archives of physical medicine and rehabilitation. 2002;83(5):613–627. doi: 10.1053/apmr.2002.32309 11994800

[48] Glaister BC, Bernatz GC, Klute GK, Orendurff MS. Video task analysis of turning during activities of daily living. Gait & posture. 2007;25(2):289–294. doi: 10.1016/j.gaitpost.2006.04.003 16730441

[49] Lowry KA, Brach JS, Nebes RD, Studenski SA, VanSwearingen JM. Contributions of cognitive function to straight-and curved-path walking in older adults. Archives of physical medicine and rehabilitation. 2012;93(5):802–807. doi: 10.1016/j.apmr.2011.12.007 22541307 PMC4878139

[50] Godi M, Nardone A, Schieppati M. Curved walking in hemiparetic patients. Journal of rehabilitation medicine. 2010;42(9):858–865. doi: 10.2340/16501977-0594 20878047

[51] Hess RJ, Brach JS, Piva SR, VanSwearingen JM. Walking skill can be assessed in older adults: validity of the Figure-of-8 Walk Test. Physical therapy. 2010;90(1):89–99. doi: 10.2522/ptj.20080121 19959654 PMC2802825

[52] Brown GL, Seethapathi N, Srinivasan M. A unified energy-optimality criterion predicts human navigation paths and speeds. Proceedings of the National Academy of Sciences. 2021;118(29):e2020327118. doi: 10.1073/pnas.2020327118 34266945 PMC8307777

[53] Segal AD, Orendurff MS, Czerniecki JM, Schoen J, Klute GK. Comparison of transtibial amputee and non-amputee biomechanics during a common turning task. Gait & posture. 2011;33(1):41–47. doi: 10.1016/j.gaitpost.2010.09.021 20974535

[54] Ventura JD, Segal AD, Klute GK, Neptune RR. Compensatory mechanisms of transtibial amputees during circular turning. Gait & posture. 2011;34(3):307–312. doi: 10.1016/j.gaitpost.2011.05.014 21696958

[55] Zarrugh M, Todd F, Ralston H. Optimization of energy expenditure during level walking. European journal of applied physiology and occupational physiology. 1974;33(4):293–306. doi: 10.1007/BF00430237 4442409

[56] Srinivasan M. Optimal speeds for walking and running, and walking on a moving walkway. Chaos: An Interdisciplinary Journal of Nonlinear Science. 2009;19(2):026112. doi: 10.1063/1.3141428 19566272

[57] Donelan JM, Kram R, et al. Mechanical and metabolic determinants of the preferred step width in human walking. Proceedings of the Royal Society of London B: Biological Sciences. 2001;268(1480):1985–1992. doi: 10.1098/rspb.2001.1761 11571044 PMC1088839

[58] Russell Esposito E, Rábago CA, Wilken J. The influence of traumatic transfemoral amputation on metabolic cost across walking speeds. Prosthetics and orthotics international. 2018;42(2):214–222. doi: 10.1177/0309364617708649 28655287

[59] Ralston HJ. Energy-speed relation and optimal speed during level walking. Internationale Zeitschrift für Angewandte Physiologie Einschliesslich Arbeitsphysiologie. 1958;17(4):277–283. 13610523 10.1007/BF00698754

[60] Minetti AE, Ardigò LP, Capodaglio EM, Saibene F. Energetics and mechanics of human walking at oscillating speeds. American Zoologist. 2001;41(2):205–210. doi: 10.1668/0003-1569(2001)041[0205:EAMOHW]2.0.CO;2

[61] Minetti AE, Gaudino P, Seminati E, Cazzola D. The cost of transport of human running is not affected, as in walking, by wide acceleration/deceleration cycles. Journal of Applied Physiology. 2013;114(4):498–503. doi: 10.1152/japplphysiol.00959.2012 23221963

[62] Minetti AE, Cazzola D, Seminati E, Giacometti M, Roi G. Skyscraper running: physiological and biomechanical profile of a novel sport activity. Scandinavian journal of medicine & science in sports. 2011;21(2):293–301. doi: 10.1111/j.1600-0838.2009.01043.x 20030780

[63] Waters R, Perry J, Antonelli D, Hislop H. Energy cost of walking of amputees: the influence of level of amputation. J Bone Joint Surg Am. 1976;58(1):42–46. doi: 10.2106/00004623-197658010-00007 1249111

[64] Esposito ER, Rodriguez KM, Ràbago CA, Wilken JM. Does unilateral transtibial amputation lead to greater metabolic demand during walking. J Rehabil Res Dev. 2014;51(8):1287–96. doi: 10.1682/JRRD.2014.06.0141 25671680

[65] Handford ML, Srinivasan M. Robotic lower limb prosthesis design through simultaneous computer optimizations of human and prosthesis costs. Scientific reports. 2016;6:19983. doi: 10.1038/srep19983 26857747 PMC4746571

[66] Collins SH, Kuo AD. Controlled energy storage and return prosthesis reduces metabolic cost of walking. Power. 2005;600:800.

[67] Herr HM, Grabowski AM. Bionic ankle–foot prosthesis normalizes walking gait for persons with leg amputation. Proceedings of the Royal Society B: Biological Sciences. 2012;279(1728):457–464. doi: 10.1098/rspb.2011.1194 21752817 PMC3234569

[68] Beck ON, Taboga P, Grabowski AM. Reduced prosthetic stiffness lowers the metabolic cost of running for athletes with bilateral transtibial amputations. Journal of Applied Physiology. 2017;122(4):976–984. doi: 10.1152/japplphysiol.00587.2016 28104752

[69] Quesada RE, Caputo JM, Collins SH. Increasing ankle push-off work with a powered prosthesis does not necessarily reduce metabolic rate for transtibial amputees. Journal of Biomechanics. 2016;49(14):3452–3459. doi: 10.1016/j.jbiomech.2016.09.015 27702444

[70] Hansen AH, Miff SC, Childress DS, Gard SA, Meier MR. Net external energy of the biologic and prosthetic ankle during gait initiation. Gait & posture. 2010;31(1):13–17. doi: 10.1016/j.gaitpost.2009.08.237 19762242

[71] Finley JM, Bastian AJ, Gottschall JS. Learning to be economical: the energy cost of walking tracks motor adaptation. The Journal of physiology. 2013;591(4):1081–1095. doi: 10.1113/jphysiol.2012.245506 23247109 PMC3591716

[72] Fey NP, Klute GK, Neptune RR. Optimization of prosthetic foot stiffness to reduce metabolic cost and intact knee loading during below-knee amputee walking: a theoretical study. Journal of biomechanical engineering. 2012;134(11):111005. doi: 10.1115/1.4007824 23387787 PMC3707817

[73] Mombaur K, Truong A, Laumond JP. From human to humanoid locomotion? an inverse optimal control approach. Autonomous robots. 2010;28(3):369–383. doi: 10.1007/s10514-009-9170-7

[74] Liu CK, Hertzmann A, Popović Z. Learning physics-based motion style with nonlinear inverse optimization. ACM Transactions on Graphics (TOG). 2005;24(3):1071–1081. doi: 10.1145/1073204.1073314

[75] Mainprice J, Hayne R, Berenson D. Predicting human reaching motion in collaborative tasks using inverse optimal control and iterative re-planning. In: 2015 IEEE International Conference on Robotics and Automation (ICRA). IEEE; 2015. p. 885–892.

[76] Bhargava R. The Jaipur Foot and the “Jaipur Prosthesis”. Indian Journal of Orthopaedics. 2019;53(1):5. doi: 10.4103/ortho.IJOrtho_162_18 30905976 PMC6394196

[77] Mali HS, Jain A, Abrams L, Sorby SA, Donahue TLH. CAD/CAE of Jaipur foot for standardized and contemporary manufacturing. Disability and Rehabilitation: Assistive Technology. 2019;. 30696308 10.1080/17483107.2018.1555865

[78] Mysore H. The Jaipur Foot: India’s Most Popular Prosthetic for Amputees Is Not the Latest in Technology, but It’s Still the Most Suitable Option for Many Patients Almost 50 Years after Its Development. IEEE pulse. 2016;7(3):30–33. doi: 10.1109/MPUL.2016.2539798 27187538

[79] Perry JA, Srinivasan M. Walking with wider steps changes foot placement control, increases kinematic variability and does not improve linear stability. Royal Society open science. 2017;4(9):160627. doi: 10.1098/rsos.160627 28989728 PMC5627068

[80] Hall P, Wilson SR. Two guidelines for bootstrap hypothesis testing. Biometrics. 1991; p. 757–762. doi: 10.2307/2532163

[81] Chernick MR. Bootstrap methods: A guide for practitioners and researchers. vol. 619. John Wiley & Sons; 2011.

[82] Miff SC, Childress DS, Gard SA, Meier MR, Hansen AH. Temporal symmetries during gait initiation and termination in nondisabled ambulators and in people with unilateral transtibial limb loss. Journal of Rehabilitation Research & Development. 2005;42(2). 15944882 10.1682/jrrd.2004.03.0038

[83] Jaegers SMHJ, Vos LDW, Rispens P, Hof AL. The Relationship Between Comfortable and Most Metabolically Efficient Walking Speed in Persons With Unilateral Above-Knee Amputation. Arch Phys Med Rehabil. 1993;74:521–525. doi: 10.1016/0003-9993(93)90117-S 8489363

[84] Genin JJ, Bastien GJ, Franck B, Detrembleur C, Willems PA. Effect of speed on the energy cost of walking in unilateral traumatic lower limb amputees. Eur J Appl Physiol. 2008;103:655–663. doi: 10.1007/s00421-008-0764-0 18478251

[85] Bobbert AC. Energy expenditure in level and grade walking. J Appl Physiol. 1960;15:1015–1021. doi: 10.1152/jappl.1960.15.6.1015

[86] Srinivasan M. Fifteen observations on the structure of energy-minimizing gaits in many simple biped models. Journal of The Royal Society Interface. 2010;8(54):74–98. doi: 10.1098/rsif.2009.0544 20542957 PMC3024815

[87] Vrieling AH, Van Keeken H, Schoppen T, Otten E, Halbertsma J, Hof A, et al. Gait initiation in lower limb amputees. Gait & posture. 2008;27(3):423–430. doi: 10.1016/j.gaitpost.2007.05.013 17624782

[88] Batten HR, McPhail SM, Mandrusiak AM, Varghese PN, Kuys SS. Gait speed as an indicator of prosthetic walking potential following lower limb amputation. Prosthetics and orthotics international. 2019;43(2):196–203. doi: 10.1177/0309364618792723 30112982

[89] Seethapathi N, Srinivasan M. Step-to-step variations in human running reveal how humans run without falling. eLife. 2019;8:e38371. doi: 10.7554/eLife.38371 30888320 PMC6424559

[90] Joshi V, Srinivasan M. A controller for walking derived from how humans recover from perturbations. Journal of The Royal Society Interface. 2019;16(157):20190027. doi: 10.1098/rsif.2019.0027 31409232 PMC6731497

[91] Carlisle RE, Kuo AD. Optimization of energy and time predicts dynamic speeds for human walking. Elife. 2023;12:e81939. doi: 10.7554/eLife.81939 36779697 PMC10030114

[92] Handford ML, Srinivasan M. Energy-optimal human walking with feedback-controlled robotic prostheses: a computational study. IEEE Transactions on Neural Systems and Rehabilitation Engineering. 2018;26(9):1773–1782. doi: 10.1109/TNSRE.2018.2858204 30040647

[93] Russell Esposito E, Miller RH. Maintenance of muscle strength retains a normal metabolic cost in simulated walking after transtibial limb loss. PLoS One. 2018;13(1):e0191310. doi: 10.1371/journal.pone.0191310 29329344 PMC5766241

[94] Nitschke M, Dorschky E, Heinrich D, Schlarb H, Eskofier BM, Koelewijn AD, et al. Efficient trajectory optimization for curved running using a 3D musculoskeletal model with implicit dynamics. Scientific reports. 2020;10(1):17655. doi: 10.1038/s41598-020-73856-w 33077752 PMC7573630

[95] Mordatch I, Wang JM, Todorov E, Koltun V. Animating human lower limbs using contact-invariant optimization. ACM Transactions on Graphics (TOG). 2013;32(6):1–8. doi: 10.1145/2508363.2508365

[96] Dudek NL, Khan OD, Lemaire ED, Marks MB, Saville L. Ambulation monitoring of transtibial amputation subjects with patient activity monitor versus pedometer. Journal of Rehabilitation Research & Development. 2008;45(4). 18712643 10.1682/jrrd.2007.05.0069

[97] Rebula JR, Ojeda LV, Adamczyk PG, Kuo AD. Measurement of foot placement and its variability with inertial sensors. Gait & posture. 2013;38(4):974–980. doi: 10.1016/j.gaitpost.2013.05.012 23810335 PMC4284057

[98] Hordacre B, Barr C, Crotty M. Use of an activity monitor and GPS device to assess community activity and participation in transtibial amputees. Sensors. 2014;14(4):5845–5859. doi: 10.3390/s140405845 24670721 PMC4029655

[99] Chambers C, Seethapathi N, Saluja R, Loeb H, Pierce SR, Bogen DK, et al. Computer vision to automatically assess infant neuromotor risk. IEEE Transactions on Neural Systems and Rehabilitation Engineering. 2020;28(11):2431–2442. doi: 10.1109/TNSRE.2020.3029121 33021933 PMC8011647

[100] Seethapathi N, Wang S, Saluja R, Blohm G, Kording KP. Movement science needs different pose tracking algorithms. arXiv preprint arXiv:190710226. 2019;.

[101] Needham L, Evans M, Cosker DP, Wade L, McGuigan PM, Bilzon JL, et al. The accuracy of several pose estimation methods for 3D joint centre localisation. Scientific reports. 2021;11(1):1–11. doi: 10.1038/s41598-021-00212-x 34667207 PMC8526586

[102] Baroudi L, Newman MW, Jackson EA, Barton K, Shorter KA, Cain SM. Estimating walking speed in the wild. Frontiers in Sports and Active Living. 2020;2:583848. doi: 10.3389/fspor.2020.583848 33345151 PMC7739717

