# Supplementary material for: Walking speeds are lower for short distance and turning locomotion: Experiments and modeling in low-cost prosthesis users
Source: PLoS One. 2024 Jan 2;19(1):e0295993. doi: 10.1371/journal.pone.0295993 (PMC10760709; doi:10.1371/journal.pone.0295993)
Supplement: S1 Appendix — S1-S5 Figs in this appendix reproduces Figs 3 and 4, with data from subjects without amputation from [17, 52] re-drawn and overlaid for a qualitative visualization, and putting the results in this manuscript in the context of the older results. (PDF) [file pone.0295993.s002.pdf]

Supplementary Appendix:
Walking speeds are lower for shorter distances and lower while turning in tighter circles: a study in low-cost prosthesis users

Nidhi Seethapathi[1,3*], Anil Kumar Jain Seethapathi[2], Manoj Srinivasan[1],

1 Mechanical and Aerospace Engineering, The Ohio State University, Columbus OH 43210, USA

2 Santokba Durlabhji Memorial Hospital, Jaipur, Rajasthan 302015, India

3 Department of Bioengineering, University of Pennsylvania, Philadelphia PA 19104, USA

* snidhi@seas.upenn.edu

Figs S1-S4 in this Supplementary Appendix reproduces Figs 2-4 from the main manuscript, with data from [1, 2] re-drawn and overlaid for a qualitative visualization, and putting the results in this manuscript in the context of the older results.

References

1. Seethapathi N, Srinivasan M. The metabolic cost of changing walking speeds is significant, implies lower optimal speeds for shorter distances, and increases daily energy estimates. Biology letters. 2015;11(9):20150486.

2. Brown GL, Seethapathi, N., Srinivasan M. A unified energy-optimality criterion predicts human navigation paths and speeds. Proc. Nat. Acad. Sci. 2021; 118 (29): e2020327118.

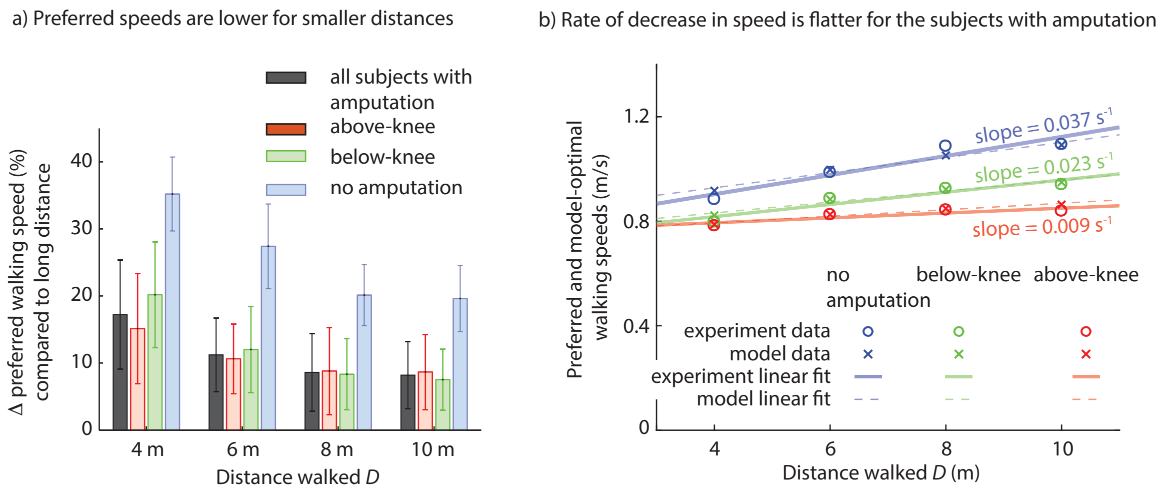

Fig S1. Decrease in preferred walking speed with distance walked for subjects with amputation. a) Amputees showed a decrease in average preferred walking speed for short distances. b) The rate of change in preferred walking speed with distance for the subjects with unilateral amputation. This is identical to Fig 2 of the main manuscript, except that data from subjects without amputation from [2] is overlaid to indicate qualitative similarity, albeit with apparent quantitative differences.

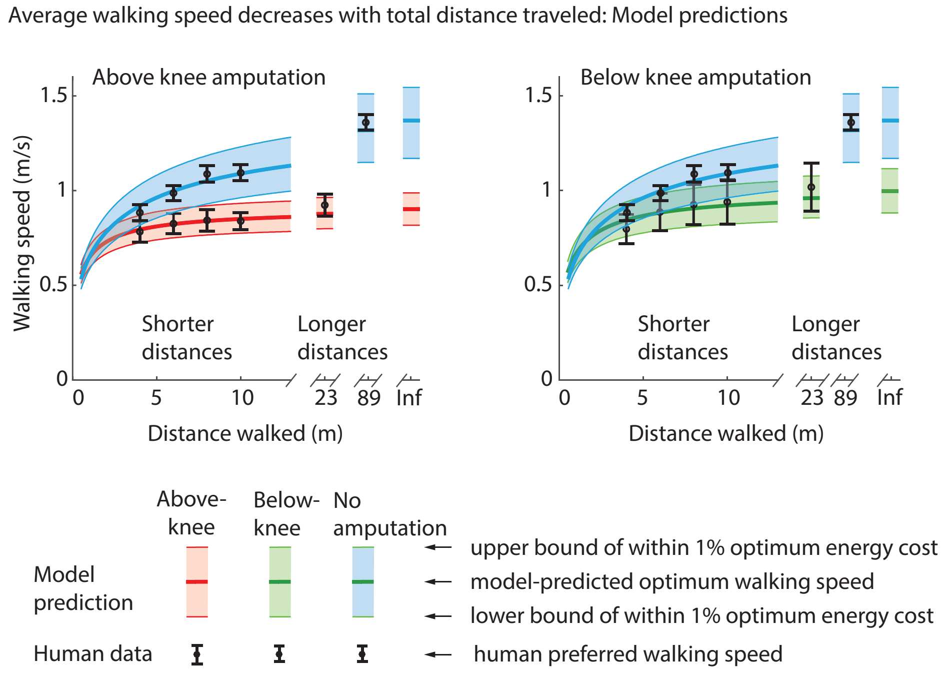

Fig S2. Minimization of total metabolic cost captures slower short-distance walking speeds. The total cost of the walking a short distance includes a term due to constant-speed cost and a changing-speed cost. We find that minimizing this total cost predicts the observed trends in changing preferred walking speed with distance. The error bars for human data represent standard errors, and the filled bands represent the set of all speeds within 1% of the energy optimal energy cost. The changing speed cost was obtained via inverse optimization, but the qualitative trends remain as long as the cost is positive. This is identical to Fig 3 of the main manuscript, except that data from subjects without amputation from [2] is overlaid to indicate qualitative similarity, albeit with apparent quantitative differences.

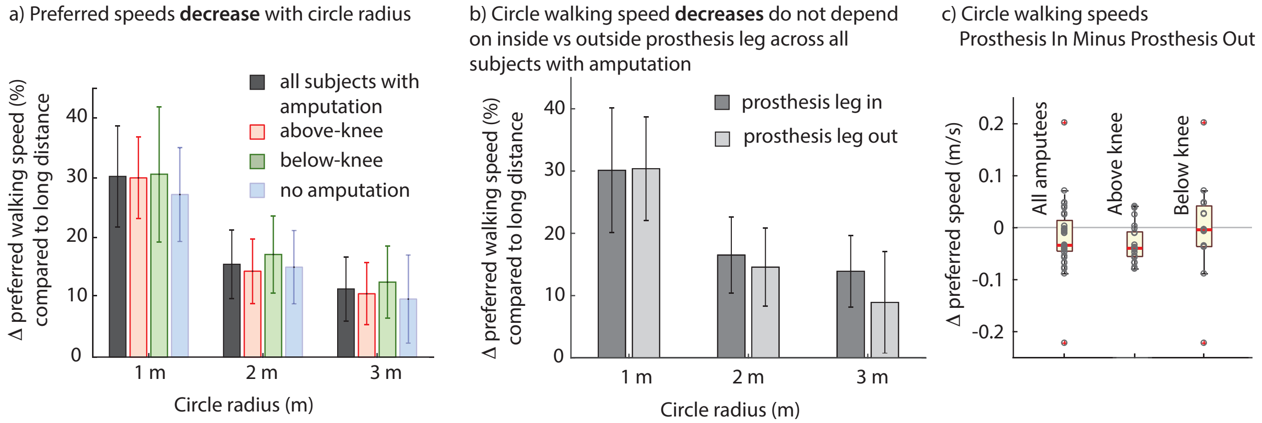

Fig S3. Preferred walking speeds for circle walking. a) The preferred walking speed for all the subjects with unilateral amputation showed a decrease with radius of the circle walked. b) Amputees, when pooled together, did not show a significant difference in preferred walking speed when walking with the prosthesis-leg inside versus outside the circle. c) Subjects with above knee amputation show a greater walking speed on average when the prosthesis leg is outside the circle. This is identical to Fig 4 of the main manuscript, except that data from subjects without amputation from [2] is overlaid to indicate qualitative similarity, albeit with apparent quantitative differences.

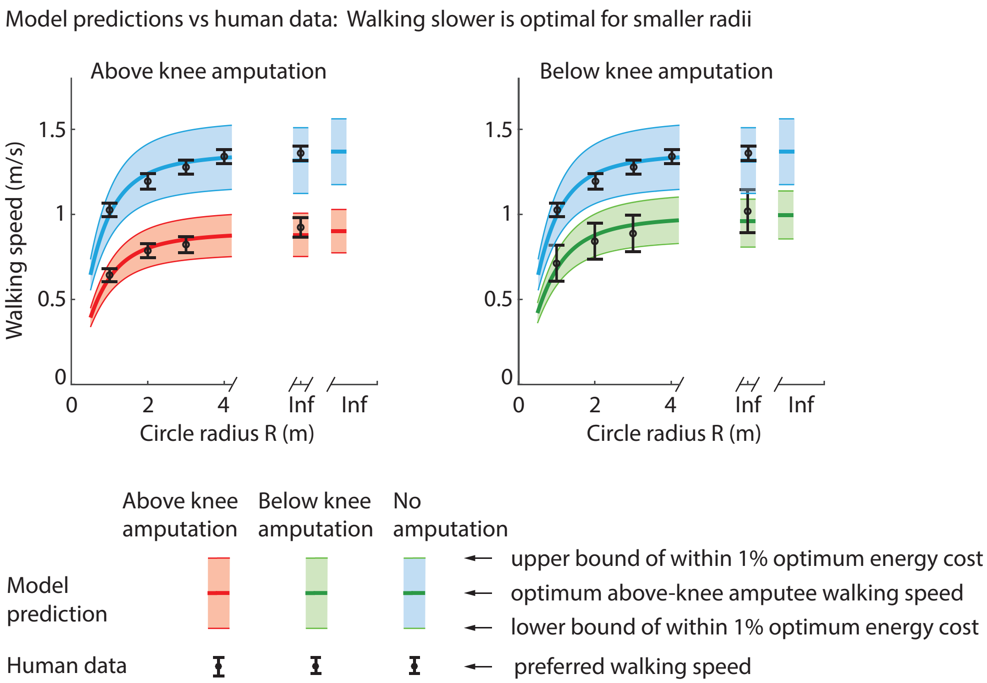

Fig S4. Optimal walking speeds for circle walking. Minimizing the energy cost of walking in a circle predicts slower walking for smaller circles. Error bars shown for the data correspond to one standard error about the mean, and these are generally within the set of all speeds within 1% of the optimal energy costs (the shaded bands shown, as in Fig S2. The cost of turning was obtained via inverse optimization, but the general monotonic trend between speed and radius will remain as long as the cost is positive. This is identical to Fig 5 of the main manuscript, except that data from subjects without amputation from [2] is overlaid to indicate qualitative similarity, albeit with apparent quantitative differences.

Walking for short distances

Model predictions vs human data: Average walking speed decreases with total distance traveled

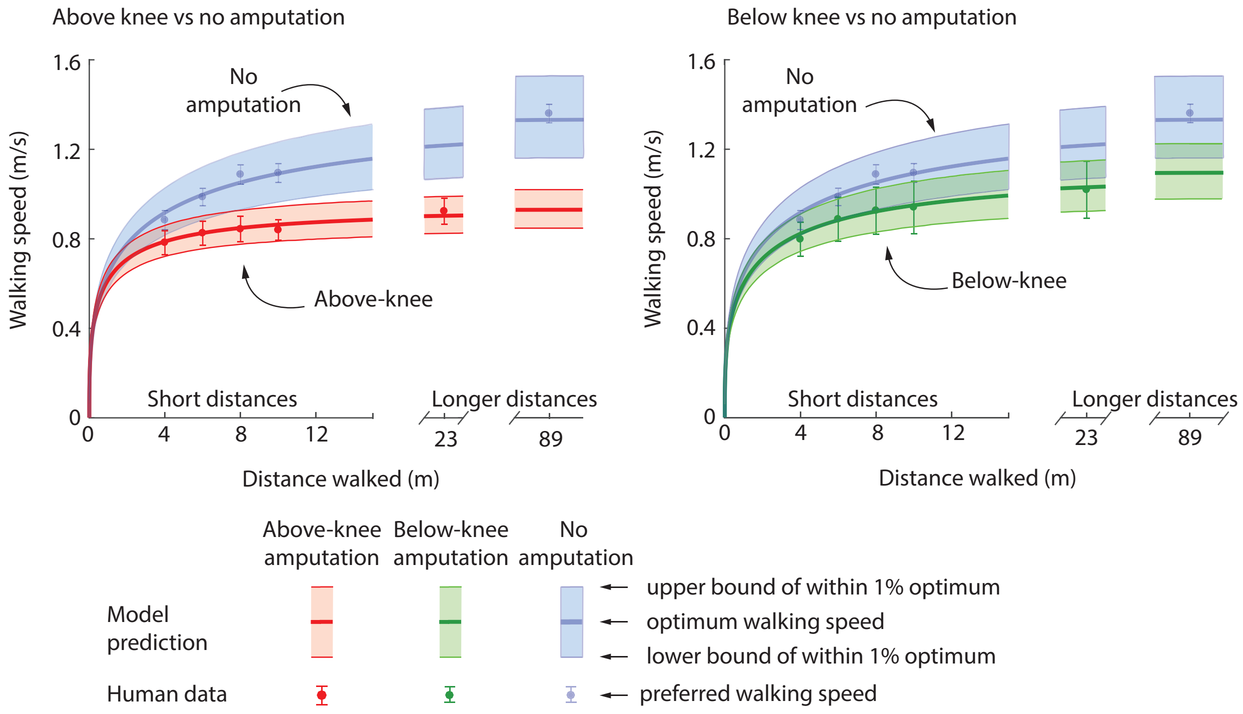

Walking in circles

Model predictions vs human data: Walking slower is optimal for smaller radii

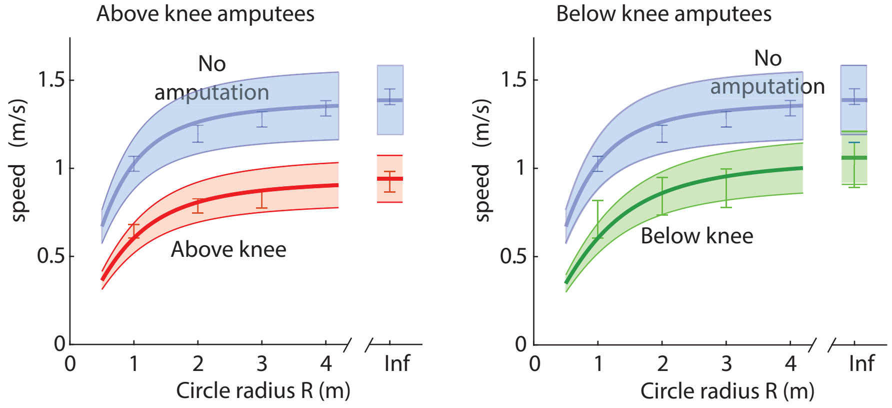

Fig S5. Another inverse optimization. This figure is based on the first version of inverse optimization, which did not change a_2 but only changed λ and b_2. For circle walking, the data for $R = \infty$ is obtained over finite straight line bout distances, 23 m in subjects with amputation and 89 m in subjects without amputation.
